# Phase I trial of split-dose induction docetaxel, cisplatin, and 5-fluorouracil (TPF) chemotherapy followed by curative surgery combined with postoperative radiotherapy in patients with locally advanced oral and oropharyngeal squamous cell cancer (TISOC-1)

**DOI:** 10.1186/1471-2407-12-483

**Published:** 2012-10-20

**Authors:** Katrin Oertel, Karin Spiegel, Harald Schmalenberg, Andreas Dietz, Georg Maschmeyer, Thomas Kuhnt, Holger Sudhoff, Thomas G Wendt, Orlando Guntinas-Lichius

**Affiliations:** 1 Department of Otorhinolaryngology, Jena University Hospital, Lessingstrasse 2, Jena, D-07740, Germany; 2 Department of Medicine II, Jena University Hospital, Jena, Germany; 3 Department of Otorhinolaryngology/Plastic Surgery, University Hospital Leipzig, Leipzig, Germany; 4 Department of Hematology, Oncology & Palliative Care, Klinikum Ernst von Bergmann, Potsdam, Germany; 5 Department of Radiation Oncology, University of Rostock, Rostock, Germany; 6 Department of Otorhinolaryngology, Head and Neck Surgery, Klinikum Bielefeld, Bielefeld, Germany; 7 Department of Radiation Oncology, Jena University Hospital, Jena, Germany

**Keywords:** Docetaxel, Cisplatin, 5-fluorouracil, Locally advanced oral cancer, Surgery, Radiotherapy

## Abstract

**Background:**

Induction chemotherapy (ICT) with docetaxel, cisplatin and fluorouracil (TPF) followed by radiotherapy is an effective treatment option for unresectable locally advanced head and neck cancer. This phase I study was designed to investigate the safety and tolerability of a split-dose TPF ICT regimen prior to surgery for locally advanced resectable oral and oropharyngeal cancer.

**Methods:**

Patients received TPF split on two dosages on day 1 and 8 per cycle for one or three 3-week cycles prior to surgery and postoperative radiotherapy or radiochemotherapy. Docetaxel was escalated in two dose levels, 40 mg/m^2^ (DL 0) and 30 mg/m^2^ (DL −1), plus 40 mg/m^2^ cisplatin and 2000 mg/m^2^ fluorouracil per week using a 3 +3 dose escalation algorithm.

**Results:**

Eighteen patients were enrolled and were eligible for toxicity and response. A maximum tolerated dose of 30 mg/m^2^ docetaxel per week was reached. The most common grade 3+ adverse event was neutropenia during ICT in 10 patients. Surgery reached R0 resection in all cases. Nine patients (50%) showed complete pathologic regression.

**Conclusions:**

A split-dose regime of TPF prior to surgery is feasible, tolerated and merits additional investigation in a phase II study with a dose of 30 mg/m docetaxel per week.

**Trial registration number:**

NCT01108042 (ClinicalTrials.gov Identifier)

## Background

The majority of patients with oral squamous cell cancer (OSCC) presents with stage III or IVa M0 disease. Surgery and/or radiotherapy are the current primary treatment modalities for locally and locoregionally advanced disease, and radiochemotherapy is an alternative for locoregionally advanced OSCC. If surgery is chosen for a locally advanced tumor, it is usually followed by adjuvant radiotherapy [1]. Moreover, among high-risk patients with resected head and neck cancer, concurrent postoperative chemotherapy and radiotherapy significantly improve the rates of local and regional control and disease-free survival [1,2]. Locoregional control seems to be improved by such a trimodal strategy using chemotherapy after surgery and concomitant with radiotherapy but distant metastasis still is a challenging problem. Especially, extended nodal involvement (≥ N2) seems to have a 30-40% risk of distant disease recurrence during follow up [3,4]. The recent update of the Meta-Analysis of Chemotherapy in Head and Neck Cancer (MACH-NC) collaborative group confirmed the significant survival benefit of adjuvant chemotherapy with a more pronounced effect if administered concurrently with radiotherapy than as induction chemotherapy (ICT) for head and neck cancer. Nevertheless, ICT had a higher impact on distant metastasis than concurrent application [5]. Focusing on oral cancer, it looks like as ICT or chemotherapy concomitantly applied with radiotherapy have the same effectiveness on overall survival [6].

The objective of ICT has been to reduce especially the risk of distant recurrence. Moreover, the addition of a taxane to platinum and 5-fluorouracil ICT (TPF; this type of ICT was not analyzed yet by the MACH-NC group) has recently been reported to be superior to platinum and 5-fluorouracil in randomized phase III studies, with increased tumor responses and overall survival (OS) [7,8]. Furthermore, TPF has also been evaluated in several phase II studies followed by radiotherapy with concomitant chemotherapy [9]. In contrast, TPF has no yet been analyzed in controlled studies as ICT prior to surgery.

The present study was therefore initiated as a phase I dose escalation trial to investigate the safety of combining TPF as ICT followed by surgery and postoperative radiotherapy or radiochemotherapy in patients with locally advanced oral cancer. The phase I design was chosen because of two reasons: First, due to several TPF-related toxic deaths in the DeLOS II trial [10], which led to discussions about the feasibility of TPF in German head and neck cancer patients. Therefore, the study protocol was designed as precautious as possible with a phase I study prior to a phase II trial. Second, but also related to the first argument, a split-dose TPF regime was chosen with the aim to reduce toxicity of TPF. Splitting the dosage of TPF has been shown to reduce the toxicity profile without reduction of the effectiveness in gastric cancer [11].

## Methods

This multicenter study was conducted at six institutions in Germany (ClinicalTrials.gov; NCT01108042). Three of them included patients in the phase I study. The study was coordinated in the Department of Otorhinolaryngology, University Hospital Jena, Germany. The protocol was centrally approved by the ethics committee for human research at the Medical Faculty, Friedrich-Schiller-University Jena, and adopted by the ethics committee of all study centers. The protocol was conformed to the principles of the Declaration of Helsinki and its subsequent amendments. All patients provided written informed consent before registration. Study treatment was given in curative intent. Patients were included from November 2009 to August 2010 and have been observed until January 2012 or death.

### Patient eligibility

Patients aged ≥18 and ≤80 years were eligible for inclusion if a histologically confirmed resectable squamous cell carcinoma of the oral cavity (excluding the lip), or of the oropharynx had been diagnosed. Tumors were judged resectable when an R0 resection was likely to be achieved. Tumors were included if they were classified as any cTcN2M0, any cTcN3M0, cT3 and cN0-1M0, cT4 and cN0-1M0 (International Union Against Cancer). Furthermore, inclusion required a Karnofsky Index (KI) ≥70% without high anesthetic risk. Adequate pulmonary, cardiac, bone marrow, hepatic and renal functions were mandatory. Exclusion criteria were distant metastatic disease; a life expectancy of <3 months; pregnancy; previous cancer disease within 5 years of study entry. Furthermore, patients were not entered in case of serious concomitant diseases or serious medical conditions; previous treatment with chemotherapy, radiotherapy or surgery for head and neck cancer; or any social situations that would have limited the compliance with study requirements. Concurrent treatments with other experimental drugs or participation in another clinical trial with any investigational drug within 30 days before study screening were also not allowed. Patients were also not enrolled in case of any contraindications against any of the chemotherapeutical drugs.

### Study design

This trial was designed as a multicenter, prospective, three-modality phase I dose escalation study. An intent-to-treat analysis was performed. The primary objective was to determine the maximum tolerated dose (MTD) of the split-dose ICT containing a combination of docetaxel (Taxotere®, Sanofi-Aventis), cisplatin and 5-fluorouracil (split TPF). Secondary objectives included acute toxicity, tumor response rate after first and last chemotherapy cycle, and rate of patients with complete treatment (ICT, surgery, postoperative radiotherapy or radiochemotherapy).

**Figure 1:**
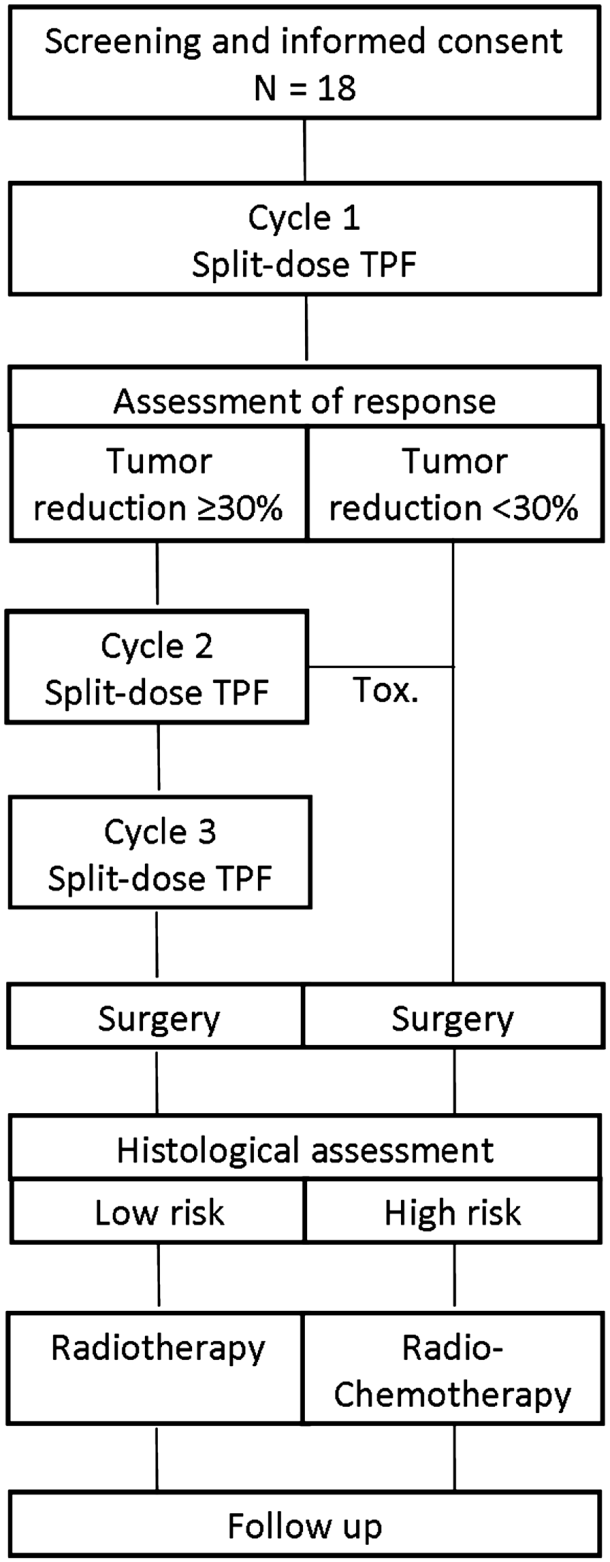
**Treatment schedule.** TPF, docetaxel+cisplatin+5-fluorouracil; Tox, determination of induction chemotherapy because of toxicity (did not occur).

Patients had to follow the treatment plan as illustrated in Figure 1. TISOC-1 started with a split-dose regime of TPF. Each chemotherapy cycle lasted 3 weeks. Chemotherapy was applied with usual premedications, appropriate antiemetics and intravenous hydration. Antibiotic prophylaxis was mandatory during the first cycle. During the first cycle, docetaxel and cisplatin were given on day 1 and 8, and 5-fluorouracil as a 24-h infusion on day 1 and 8. Tumor response was evaluated on day 21 using a clinical examination with endoscopy of the primary tumor. Additionally, a CT or MRI of the neck with tumor volumetry was performed. Tumor response to chemotherapy was defined by the RECIST criteria and as a reduction of the tumor volume ≥ 30%. [12] Complete remission (CR) was defined as complete disappearance of primary tumor and neck metastasis. Partial response (PR) was defined as mentioned above but without complete disappearance. Progressive disease (PD) was defined as enlargement of tumor volume ≥ 20% or new tumor manifestations. Stable disease (SD) was fulfilled for tumors with enlargement <20% to reduction < 30%. Responders were patients with CR or PR. All responders received two more cycles of split-TPF, i.e. responders underwent surgery after three cycles and non-responders after one cycle of ICT. Treatment cycles were repeated if the absolute neutrophil count (ANC) was ≥ 1.000/mm^3^, platelet count was ≥ 80.000/mm^3,^ neurotoxicity ≤ grade 1, hand-foot-syndrome ≤ grade 1, and adequate kidney and liver function. If these criteria had not been recovered after a 2-week delay, chemotherapy was continued with a 70% reduction after recovery of the blood parameters. Prophylactic use of a granulocyte- colony stimulating factor before the second and third cycle was recommended if a patient had a febrile neutropenia or a delayed recovery of ANC.

Docetaxel and cisplatin were escalated at different levels in successive cohorts as a classic 3 + 3 dose escalation design. The planned dose levels for docetaxel were 40 and 50 mg/m^2^ and for cisplatin also 40 and 50 mg/m^2^ on days 1, 8 (cycle 1), 22, 29 (cycle 2), and 43, 50 (cycle 3). The dose of 5-fluorouracil was fixed to 2000 mg/m^2^ as 24-h infusion on days 1, 8 (cycle 1), 22, 29 (cycle 2), and 43, 50 (cycle 3). Planned levels of the dose escalation were as follows: Level −1: 30 mg/m^2^ docetaxel, 40 mg/m^2^ cisplatin, 2000 mg/m^2^ 5-fluorouracil. Level 0: 40 mg/m^2^ docetaxel, 40 mg/m^2^ cisplatin, 2000 mg/m^2^ 5-fluorouracil. Level 1: 50 mg/m^2^ docetaxel, 40 mg/m^2^ cisplatin, 2000 mg/m^2^ 5-fluorouracil. Level 2: 50 mg/m^2^ docetaxel, 50 mg/m^2^ cisplatin, 2000 mg/m^2^ 5-fluorouracil. Escalation started with level 0 and ended with −1. The levels 1 and 2 were not reached.

Acute toxicity was assessed using the National Cancer Institute’s Common Toxicity Criteria (NCICTC) version 3.0. Dose-limiting toxicity (DLT) was defined as neutropenia grade 3/4. Three patients were entered in dose escalation level 0. In case of one or two DLT out of three patients at any dose level, three additional patients had to be entered at the same dose escalation level. If no additional DLTs occurred, the dose escalation proceeded to level 1 (further escalation from level 1 to level 2 with the same rules). In case of three or four DLTs in six patients at a particular dose level, three additional patients had to be entered at the same dose escalation level 0. In case of three in three patients, respectively, five or six DLTs in six patients at a particular dose level, the escalation was reduced to level −1. Six more patients had to be entered at this escalation level. The MTD was defined as the dose level of docetaxel and cisplatin (below the maximal administered dose) at which none of nine patients experienced a DLT. Furthermore, at least nine patients at the final dose level had to pass through the complete protocol before giving a recommendation for the phase II study. If the MTD was not reached, the highest tested dose of cisplatin (50 mg/m^2^) and docetaxel (50 mg/m^2^) were to be recommended for future studies. In addition to DLT, all other therapy dropouts related to toxicity as well as all other non-hematologic toxicity ≥ grade 3 were evaluated.

Surgery was recommended in-between 3–5 weeks after last chemotherapy. Surgery was performed within the original tumor margins prior to ICT. Each study center could decide individually the type of surgery (transoral laser surgery, classical transoral resection, open surgery, usage of local or free flaps). Ipsilateral neck dissection was obligatory. For tumors crossing the midline a contralateral neck dissection was recommended. Start of postoperative radiotherapy was recommended not later than 8 weeks after last surgery. Criteria for high risk patients were: R1 resection, resection margins < 5mm, extracapsular spreading, perineural tumor growth, vascular tumor embolus, lymph node metastasis in level IV or V, > 2 positive lymph nodes. All other patients were classified as low risk patients. All tumors were tested for human papillomavirus (HPV) DNA by PCR as described previously. [13] Postoperative radiotherapy was applied either as 3 D conformal or with intensity modulated radiotherapy (IMRT). Total dose to the target volume at the previous primary and at levels with extracapsular lymph nodes were scheduled to receive 64 Gy, involved lymph node levels without risk factors 60 Gy and uninvolved levels 50 Gy with 2 Gy per fraction. IMRT was specified according to the RTOG 0022 protocol. High risk patients received radiochemotherapy with cisplatin (20 mg/m^2^) five times in week 1 and week 5 of radiotherapy.

### Study evaluations

All patients who received at least one dose of ICT were evaluated for safety and response. Maximal three weeks before the initiation of the therapy, all patients had to undergo a full clinical examination including head and neck and dental examination as well as hematological and biochemical assessments. All patients received a panendoscopy with tattooing of the tumor margins, a sonography of neck and abdomen, chest x-ray or computed tomography (CT) of the thorax, CT or magnetic resonance imaging (MRI) of the neck. Every treatment week included a clinical examination and evaluations of KI/ECOG and toxicity. Hematologic and biochemical assessments were carried out at least once weekly during ICT, always before starting a new cycle, and during postoperative radiochemotherapy. An audiogram was performed after each cycle of chemotherapy. Swallowing function was assessed using the penetration-aspiration-scale and Prosiegel’s grading [14,15]. Directly after completion of therapy, all assessments were repeated. Disease progression, toxicity and survival were subsequently evaluated every 3 months for 1 year and at 6-month intervals until year 2 for death or loss to follow-up.

## Results

### Patient demographics

Eighteen patients with a median age of 60 years were enrolled. The median follow up time was 6.1 months (range, 3.6 – 7.2 months). Baseline patient and tumor characteristics are given in Tables 1 and 2. Two thirds were male and one third female patients. The tumor sites cavity of the mouth and oropharynx were nearly equally represented. All but two tumors were classified stage IVa. Tumor stages cT2-4 were equally distributed. Four of five patients had neck metastasis. Three patients (17%) had HPV positive tumors. All but one patient completed the trimodal therapy. One patient refused postoperative radiotherapy. All 18 patients were assessable for toxicity and response.

**Table 1:** Patient demographics

**Characteristic**	**Number of patients (%), *N *= 18**	
Gender		
Male	12	(67)
Female	6	(33)
Age (years)		
Median	60	
Range	49 -74	
ECOG performance		
Grade 0	16	(89)
Grade 1	2	(11)
Body mass index (kg/m^2^)		
Median	21	
Range	20 - 24	
Primary site		
Cavity of the mouth	8	(44)
Oropharynx	10	(56)
Tumor volume (ml) of primary tumor		
Median	25	
Range	3 - 84	
AJCC stage		
III	2	(11)
IVA	16	(89)
HPV status		
Negative	15	(83)
Positive	3	(17)

ECOG, Eastern Cooperative Oncology Group; AJCC, American Joint Committee on Cancer; HPV, Human papilloma virus.

**Table 2:** TNM staging

	**T2**	**T3**	**T4a**	**Total**
N0	0	2	2	4
N1	0	0	1	1
N2a	0	1	1	2
N2b	2	1	0	3
N2c	4	2	2	8
Total	6	6	6	18

TNM, tumor–node–metastasis.

### Induction chemotherapy administered and DLT

Escalation started with level 0 and ended with −1. The levels 1 and 2 were not reached. Twelve patients were treated at dose level 0 and six at dose level −1. Seven DLT occurred in dose level 0 and one in dose level −1 (Table 3**)**. DLT occurred as neutropenia grade 4 in two cases and as neutropenia grade 3 in 5 cases. Five of the seven patients with a DLT received granulocyte colony-stimulating factor support. DLT and consequent delayed recovery was the reason to stop ICT in three patients after one cycle although PR or CR was observed. All patients who received a second cycle of ICT could also finish the third cycle of chemotherapy. ICT was delivered over a median period of 49.5 days (range, 1 – 61 days). A majority of the patients received the full course of induction treatment planned per protocol, in terms of number of chemotherapy doses and dose-intensity (Table 3).

**Table 3:** Administration of induction chemotherapy

**Induction chemotherapy parameter**	**Number of patients (%), *N *= 18**					
Dose level						
0	12	(67)				
-1	6	(33)				
DLT	8	(44)				
in dose level 0	7	(38)				
in dose level -1	1	(6)				
Number of doses* of TPF (Median, range)	6, 1 - 6					
Number of patients completed maximal	level 0		level -1		all patients	
number of cycles induction chemotherapy	*N* = 12		*N* = 6		*N* = 18	
3 cycles	7	(58)	5	(83)	12	(67)
2 cycles	7	(58)	5	(83)	12	(67)
1 cycle**	5	(42)	1	(17)	6	(33)
Dose intensity***, overall (Median, range)						
Docetaxel	333.8, 78.2 – 474					
Cisplatin	375.3, 78.2 – 474					
5-Fluorouracil	10020, 3211 - 11851					
Dose density**** per cycle	Week 1			Week 2		
Dose density, cycle 1 (Median, range)						
Docetaxel	72.6, 45.2 – 80.5			71.3, 45 – 80		
Cisplatin	73.96, 60.3 – 80.5			73, 45.3 – 80		
5-Fluorouracil	3708, 3016 - 4025			3680, 3002 - 4000		
Dose density, cycle 2 (Median, range)						
Docetaxel	65.9, 45 – 79.2			65.8, 44.8 – 79.2		
Cisplatin	70.8, 44.9 – 79.2			71, 44.8 – 79.2		
5-Fluorouracil	3572, 2990 - 3960			3550, 2992 – 3960		
Dose density, cycle 3 (Median, range)						
Docetaxel	66.2, 46.9 – 78.6			60.1, 36.1 – 78.6		
Cisplatin	71.3, 44.9 – 78.6			69.5, 45.1 – 78.6		
5-Fluorouracil	3546, 2392 - 3931			3482, 2404 - 3931		
Number of patients with dose reductions	4			(22)		

### Response to induction chemotherapy

Fifteen patients (83%) showed at least a PR after one cycle of ICT. Twelve of these fifteen patients could receive three cycles of chemotherapy and finally ended ICT in 3 cases with CR and in 9 cases with PR (Table 4). Histopathologic examination after curative tumor surgery revealed a pCR of the primary tumor in 9 patients (50%) and pCR of primary tumor and neck metastasis in 5 patients (36%). All three HPV positive tumors revealed a pCR of primary tumor and neck metastasis (ypT0ypN0). The rate of pCR was higher after 3 cycles than after 1 cycle of chemotherapy. The pCR response rates were not different in dose level 0 and level −1.

**Table 4:** Response to induction chemotherapy

**Assessment parameter**	**Number of patients (%),*N *=18**					
Clinicoradiologic assessment after first cycle of induction chemotherapy	HPV-		HPV+		all patients	
	*N* =15		*N* = 3		*N* =18	
CR	1	(7)	1	(33)	2	(11)
PR	11	(73)	2	(67)	13	(72)
SD	2	(13)			2	(11)
PD	1				1	(6)
Clinicoradiologic assessment after third cycle of induction chemotherapy						
CR	3	(25)				
PR	9	(75)				
Histopathologic result	1 cycle		3 cycles		all patients	
pCR of primary tumor	1	(6)	8	(44)	9	(50)
pCR of neck metastasis*	2	(14)	4	(29)	6	(33)
pCR of primary and neck*	1	(7)	4	(29)	5	(27)
Histopathologic result	level 0		level -1		all patients	
	*N* =12		*N* = 6		*N* =18	
pCR of primary tumor	6	(50)	3	(50)	9	(50)
pCR of neck metastasis*	5	(42)	1	(17)	6	(33)
pCR of primary and neck*	4	(33)	1	(17)	5	(27)
yTNM	HPV-		HPV+		all patients	
	*N* =15		*N* = 3		*N* =18	
ypT0ypN0	3	(20)	3	(100)	6	(33)
ypT0ypN1	1	(7)			1	(6)
ypT0ypN2	2	(13)			2	(11)
ypT1ypN0	2	(13)			2	(11)
ypT1ypN1	2	(13)			2	(11)
ypT1ypN2	1	(7)			1	(6)
ypT2ypN2	1	(7)			1	(6)
ypT3ypN0	1	(7)			1	(6)
ypT4ypN0	1	(7)			1	(6)
ypT4ypN2	1	(7)			1	(6)
Histopathologic risk classification						
Low risk	14	(78)				
High risk	4	(22)				

*4 patients presented with cN0, therefore only 14 patients went into this calculation (in level 0: 1 patient; in level −1: 3 patients).

### Toxicity

The main toxicity occurred during ICT (Table 5) in terms of neutropenia grade 3 and 4. Of note, prophylactic use of antibiotics but not of granulocyte colony-stimulating factor was mandatory. One renal failure occurred that recovered completely. The leading complaint after surgery was dysphagia and mucositis at the end of postoperative radiotherapy. No treatment-related deaths were observed.

**Table 5:** Grade 3 and 4 adverse events associated with the induction chemotherapy

**Toxicity**	**Level 0**	**(%)**	**Level −1**	**(%)**	**All patients**	**(%)**
	***N *= 12**		***N *= 6**		***N *=18**	
Hematologic						
Neutropenia	9	(75)	1	(17)	10	(56)
Leukopenia	-	-	1	(17)	1	(6)
Anemia	1	(8)	-	-	1	(6)
Non-hematologic						
Diarrhea	1	(8)	-	-	1	(6)
Dysphagia	1	(8)	-	-	1	(6)
Mucositis	4	(33)	1	(17)	5	(28)
Hyperkalemia	-	-	1	(17)	1	(6)
Hyponatremia	1	(8)	1	(17)	2	(11)
Renal failure	1	(8)	-	-	1	(6)

### Surgery

Surgery was performed after a median time of 34 days (range, 14 – 50 days) after end of ICT. Transoral surgery of the primary tumor was performed in 12 cases (67%; 11 cases with laser surgery), open surgery in 6 cases (33%, 4 with local or free flap for reconstruction). Neck dissection was performed in all but one case on both sides. Complete tumor resection with microscopically clear margins (R0 resection) was achieved in all patients. As major surgical complications blood loss of more than 500 ml was observed in three cases, a chyle fistula in one case, and a wound infection on another case.

### Postoperative radiotherapy or radiochemotherapy

Radiotherapy or radiochemotherapy was performed after a median time of 45.5 days (range, 21 – 63 days) after surgery. Radiotherapy or radiochemotherapy lasted a median time of 44 days (range, 39 – 72 days). Median complete trimodal treatment time was 5.3 months (range, 3.3 – 6.5 months). One patient with ypT0ypN0M0 refused radiotherapy. Four patients were classified as high risk (3 patients in dose level 0 and 1 patient in level −1), but two of them could not receive adjuvant chemotherapy because of general condition and comorbidity. Minor deviations from the protocol were seen in six patients (interruption of radiotherapy because of technical reasons, or because of a holiday). Concomitant chemotherapy with cisplatin was performed in both cases without significant events.

### Functional assessments of swallowing

Prior to therapy swallowing was normal in all patients (Rosenbek score 1; Prosiegel score 0). After ICT remained normal in all but two patients (median Rosenbek score: 1; range, 1 – 2; median Prosiegel score 0; range, 0 – 3). After surgery significant dysphagia occurred in four patients (median Rosenbek score: 1; range, 1 – 6; median Prosiegel score 1; range, 0 – 6). After postoperative radiotherapy at the end of the complete trimodal therapy, swallowing function already improved in the before affected patients and was normal in most patients (median Rosenbek score: 1; range, 1 – 4; median Prosiegel score 0; range, 0 – 5).

## Discussion

The present trial showed that ICT with a split-dose regime of TPF was safe and did not jeopardize curative surgery of primary head and neck cancer and neck metastasis as well as postoperative radiotherapy or radiochemotherapy. The feasibility of such a complex trimodal treatment approach for patients with locally advanced head and neck cancer was proven. The primary objective, the determination of MTD of the split-dose regime, was achieved: a split-dose regime of 30 mg/m^2^ docetaxel, 40 mg/m^2^ cisplatin and 2000 mg/m^2^ 5-fluorouracil was recommended for the subsequent and still ongoing phase II study.

The regimen was moderately well tolerated. The rate of DLT in form of grade 3 and 4 neutropenia was not higher than expected. Using the presented split-dose regimen neutropenia grade 3 and 4 was observed in 56% of the cases. In comparison, the two large phase III studies with a standard TPF regimen observed this hematologic toxicity in 38% and 76% of the cases, respectively [7,8]. Safety is a major concern when subjecting patients with head and neck cancer to ICT prior to a standard therapy consisting of curative surgery and postoperative radiotherapy. It is very important to emphasize that other relevant acute toxicities were very rare in the present study as it was also shown for a comparable split-dose strategy in gastric cancer [11]. In turn, the acceptable acute toxicity is the reason that treatment delays were a rare event. Surgery was performed 14 – 50 days after end of ICT. Hence, ICT causes a treatment delay in comparison to standard therapy, i.e. to perform the tumor surgery directly without ICT. The prognostic role of a treatment delay on the outcome of head and neck cancer is controversially discussed. There are studies showing a negative effect of a delay on the survival but other studies not [16]. We have to await the follow-up data to see if the time interval between end of ICT and surgery has influence on the final outcome. Finally, the satisfactory treatment tolerance caused that all but one patient finished the complete protocol. Most of the patients ended therapy with nearly normal swallowing function. This might be a benefit in comparison to surgery and postoperative radiotherapy in combination with postoperative chemotherapy. We conclude that the strategy with a split-dose regimen of TPF has favorable toxicity profile using it if ICT is planned prior to surgery of a locally advanced head and neck cancer. Alternatively, it might be also a feasible strategy for further clinical trials to omit fluorouracil. This might allow higher dosages of docetaxel [17].

The presented neoadjuvant setting with subsequent surgery allowed an evaluation of the pathologic response to the split-dose TPF regime. To our knowledge, we present the first pathologic response data for TPF as it was used so far mainly prior to radiotherapy or radiochemotherapy. In such a setting, the clinicoradiologic overall response rate to TPF for head and neck cancer is about 70% (complete response: 9-17%; [7,8]. The present study limited to oral and oropharyngeal cancer gives comparable results with a clinicoradiologic overall response of 83% (complete response: 16%). Interestingly, histopathologic results revealed a complete tumor regression of the primary tumor in 50% of the patients and for primary and neck metastasis in 27% of the cases. It seems that the clinicoradiologic assessment underestimates the complete tumor response. The HPV positive tumors showed all a complete histopathologic tumor response. Nevertheless, at least two third of the patients (in our study all HPV negative) present viable tumor cells after ICT and neck metastasis and primary tumor in the same patients must not respond equally. The tumor cells remnants in the primary seem to be randomly distributed in the necrotic former tumor area (data not shown). Therefore, it has to be emphasized that tumor surgery has to be orientated on the initial extension of the tumor. Surgery seems to be an effective therapeutical option to treat such tumor remnants as R0 resection was achieved in all cases. Downstaging of the primary oral and oropharyngeal cancer for surgery is not allowed after ICT with split-dose TPF. On the other hand, ICT using split-dose TPF plus surgery resulted in a low risk postoperative constellation in 3 out of 4 patients, i.e. this presented ICT strategy seem to decrease the rate of high-risk constellations after surgery as we would expect about half of the patients with high risk post-surgical constellation after standard surgery without ICT [18].

The results of the two large phase III studies (TAX 323 and TAX 324; [7,8] led to a renaissance of ICT prior to radiotherapy or radiochemotherapy and there is an ongoing debate of the effectivity in comparison to concurrent radiochemotherapy. The present study and the meanwhile initiated phase II study adopt the idea to a trimodal concept including surgery. This is the first ongoing phase II trial including TPF as ICT prior to surgery in a trimodal therapy concept. There are two recent large randomized studies investigating the outcome of ICT prior to surgery. The one from the GETTEC group is showing that overall survival after ICT with PF prior to surgery and postoperative radiotherapy is better than after surgery and postoperative radiotherapy alone [19]. In this study, PF induction achieved in 56% of patients an objective response. This is accordance to large TAX 323 and 324 trials showing a much less response for PF than for TPF [7,8]. Unfortunately, the GETTEC study did not present data on the histopathologic response. Hence, a comparison of the histopathologic response effect after PF in the GETTEC study versus the effect of TPF in the present study is not possible. In contrast to the GETTEC trial, the addition of primary chemotherapy with PF to standard surgery was unable to improve survival in the other recent randomized trial from Italy [17]. The Italian study was restricted to cancer of the oral cavity. The objective response rate restricted to this tumor location was higher with 82%. A pathologic complete response of the primary tumor was observed in 27% of patients, i.e. half as effective like the TPF regimen in the present study. Unfortunately, the publication of the Italian trial does not give clear data on the pathologic complete response of the neck. Hence, a more detailed comparison is not possible. In the Italian trial only high-risk patients received postoperative radiotherapy. As one third of patients in the ICT group received postoperative radiotherapy, it could be concluded that PF is also able to decrease the number of high risk patients. But the present study with only a quarter of high risk patients gives some evidence that TPF is more effective than PF to decrease the number of postoperative high risk patients.

## Conclusions

We report here on the first dose escalation trial of docetaxel in a TPF regimen as part of a three-modality regimen using ICT prior to surgery. Using a split-dose application mode, TPF with 30 mg/m^2^ docetaxel, 40 mg/m^2^ cisplatin and 2000 mg/m^2^ fluorouracil can be safely combined with surgery and postoperative radiotherapy or radiochemotherapy. The good feasibility of the trimodal concept offers evidence for extending the study into a phase II trial, which is currently ongoing.

## Competing interests

This work was supported by Sanofi-Aventis. Sanofi-Aventis had no influence on the study performance data analysis. The company also had no influence on the editing and reviewing of this manuscript. No author has actual or potential conflict of interest in accordance with the journal policies.

## Authors’ contributions

OGL, AD, GM, TK and TGW contributed to the study design, clinical study procedures, data interpretation and preparation of the final manuscript. KO and KS contributed to patient recruitment and clinical study procedures. HSch and HS contributed to clinical study procedures, data interpretation and preparation of the final manuscript. All authors read and approved the final manuscript.

## Pre-publication history

The pre-publication history for this paper can be accessed here:

http://www.biomedcentral.com/1471-2407/12/483/prepub
